# Atypical Sjögren-Like Presentation as a Paraneoplastic Manifestation of Metastatic Parotid Adenocarcinoma

**DOI:** 10.7759/cureus.113725

**Published:** 2026-07-31

**Authors:** Carlos Berrocal, Isabella Libreros Mejia, Raúl Vallejo-Serna, Bryan David Zamora Segura, Jose Luis Pineda Bocanegra, Gisella Barrios Torrejano, Mariana Ariza Martinez, Nilton Adrián Arias Molina, Lena Isabel Barrera Vergara

**Affiliations:** 1 Internal Medicine, Universidad del Valle, Cali, COL; 2 General Medicine, Universidad Libre, Cali, COL; 3 Internal Medicine, See outside Hospital Universitario del Valle Evaristo García ESE, Cali, COL; 4 Internal Medicine, Hospital San Jerónimo ESE, Montería, COL; 5 Internal Medicine, Universidad del Sinú, Monteria, COL; 6 General Medicine, Clínica Materno Infantil Casa del Niño, Montería, COL; 7 General Medicine, Clínica IMAT Oncomédica Auna, Montería, COL; 8 Pathology, Universidad del Valle, Cali, COL; 9 Internal Medicine, Centro para el Desarrollo y Evaluación de Políticas y Tecnología en Salud Pública (CEDETES), Cali, COL; 10 Internal Medicine, Prevención y Control de la Enfermedad Crónica (PRECEC), Cali, COL; 11 Internal Medicine, Faculty of Health Sciences, Universidad del Valle, Cali, COL

**Keywords:** anti-ssa/ro antibodies, paraneoplastic syndrome, parotid neoplasms, polyserositis, salivary gland tumors, sjögren syndrome

## Abstract

Paraneoplastic Sjögren-like syndrome is a rare extraglandular manifestation of occult malignancy that can closely mimic primary autoimmune disease, leading to delayed diagnosis of the underlying neoplasm. While most reported cases are associated with hematological malignancies, its occurrence in the context of solid tumors, particularly salivary gland carcinomas, is exceptionally rare and poorly characterized. We report a 43-year-old male presenting with progressive dyspnea, constitutional symptoms, and severe polyserositis, initially suggestive of primary Sjögren's syndrome (SS) based on objective sicca findings, including severely reduced lacrimal secretion on the Schirmer test and clinically observed asialia, in conjunction with positive anti-SSA antibodies. Imaging showed persistent pulmonary lesions and pleural effusions, while bronchoalveolar lavage revealed markedly elevated galactomannan levels, suggesting probable pulmonary aspergillosis. Despite immunosuppressive therapy, no clinical improvement was observed. Further evaluation, including surgical biopsy, demonstrated metastatic adenocarcinoma of probable salivary gland origin. The final diagnosis was stage IV parotid adenocarcinoma with pulmonary metastases and paraneoplastic Sjögren-like syndrome. The patient received one cycle of chemotherapy but subsequently deteriorated and died. Paraneoplastic Sjögren-like syndrome is exceptionally rare and characterized by heterogeneous clinical presentations, often lacking classic histopathological findings. Red flags such as rapid progression, systemic involvement, and poor response to standard therapy should prompt evaluation for occult malignancy. This case highlights the importance of recognizing atypical Sjögren-like presentations as a probable paraneoplastic phenomenon. Early identification of warning signs may facilitate timely diagnosis of underlying malignancy and impact clinical outcomes.

## Introduction

Sjögren’s syndrome (SS) is a systemic autoimmune disease characterized by dysfunction of exocrine glands and the presence of autoantibodies, with a clinical spectrum that may include extraglandular involvement and an increased risk of lymphoma [1,2]. Although its association with hematologic malignancies is well established, Sjögren-like presentations as a paraneoplastic phenomenon in solid tumors are exceptional and represent a significant diagnostic challenge. These forms may mimic primary SS but often differ in their clinical behavior, course, and response to treatment, requiring a high index of suspicion. Currently, there are no validated diagnostic criteria for paraneoplastic Sjögren-like syndrome, and its diagnosis relies on clinicopathological correlation. We present a case of paraneoplastic Sjögren-like syndrome associated with metastatic parotid adenocarcinoma, with atypical systemic manifestations that hindered its initial recognition.

## Case presentation

A 43-year-old male with no known past medical history, an ex-smoker with a 23-year history of active smoking (two to four cigarettes/day; pack-year index: 3.8), having stopped three months prior to presentation, initially presented to a secondary care institution with a 15-day history of dry cough, progressive dyspnea on minimal exertion, and non-cardiac chest pain. Imaging studies revealed consolidation in the right upper lobe associated with bilateral pleural effusions, and he was treated as pneumonia with ampicillin/sulbactam plus clarithromycin for six days. Due to the lack of clinical improvement, antibiotic therapy was escalated to piperacillin/tazobactam. During hospitalization, he developed azotemia (blood urea nitrogen (BUN) 50 mg/dL, creatinine 2.6 mg/dL), oligoanuria, and lower-extremity edema, prompting urgent referral. Subsequently, he experienced worsening respiratory status and was transferred to our institution as a life-threatening emergency.

On admission, review of systems revealed unintentional weight loss of 14% (from 65 kg to 56 kg in 25 days). On physical examination, the patient appeared in poor general condition, tachypneic, and somnolent, with the following vital signs: blood pressure 112/86 mmHg, heart rate 114 bpm, respiratory rate 26 breaths per minute, oxygen saturation 97% on a non-rebreather mask at 10 L/min, and temperature 36°C. Physical findings included bilateral jugular venous distension, decreased breath sounds at both lung bases, abdominal distension with central ascites, grade II lower-extremity edema, delayed capillary refill (four to five seconds), and signs of dehydration.

Laboratory evaluation showed a negative infectious profile, while rheumatologic testing revealed positive antinuclear antibodies (ANA), detected by indirect immunofluorescence (IIF) on HEp-2 cells at a titer of 1:160 with a fine speckled nuclear pattern (ICAP-AC4). Extractable nuclear antigens (ENAs) were quantified by enzyme-linked immunosorbent assay (ELISA), revealing markedly elevated anti-SSA (Ro) antibodies (>200 U/mL), with negative anti-SSB (La), anti-RNP/Sm, anti-Sm, and anti-Scl-70 (see Table 1).

**Table 1:** Laboratory and serological findings BUN: blood urea nitrogen; ANA: antinuclear antibodies; ICAP: International Consensus on ANA Patterns; anti-dsDNA: anti-double-stranded DNA antibodies; anti-SS-B (La): anti-Sjögren's-syndrome-related antigen B antibodies; anti-RNP/Sm: anti-ribonucleoprotein/Smith antibodies; anti-SS-A (Ro): anti-Sjögren's-syndrome-related antigen A antibodies; anti-Sm: anti-Smith antibodies; C3: complement component 3; C4: complement component 4; C-ANCA/P-ANCA: cytoplasmic/perinuclear antineutrophil cytoplasmic antibodies; RF: rheumatoid factor

Test	Result	Reference range
Serum creatinine (mg/dL)	2.08	0.6-1.2
Blood urea nitrogen (BUN) (mg/dL)	57	7-20
Albumin (g/dL)	3.24	3.5-5.0
Sodium (mmol/L)	134	135-145
Potassium (mmol/L)	4.02	3.5-5.0
Phosphorus (mg/dL)	5.4	2.5-4.5
Ionized calcium (mmol/L)	1.07	1.18-1.32
Urine protein/creatinine ratio (mg/g)	241	<150
Antinuclear antibodies (ANA)	1:160, fine speckled nuclear pattern (ICAP-AC4)	<1:80: negative / >1:80: positive
Anti-dsDNA	Negative	Negative
Anti-SS-B (La) (U/mL)	11.5	<20: negative / >20: positive
Anti-RNP/Sm (U/mL)	1.5	<20: negative / >20: positive
Anti-SS-A (Ro) (U/mL)	>200	<20: negative / >20: positive
Anti-Sm (U/mL)	0.4	<20: negative / >20: positive
Complement C3 (mg/dL)	115	90-180
Complement C4 (mg/dL)	9.09	10-40
C-ANCA and P-ANCA	Negative	Negative
Anti-Scl-70 (U/mL)	4.3	Normal: <15
Rheumatoid factor (IU/mL)	4.87	Normal: 0-11.99

**Figure 1:**
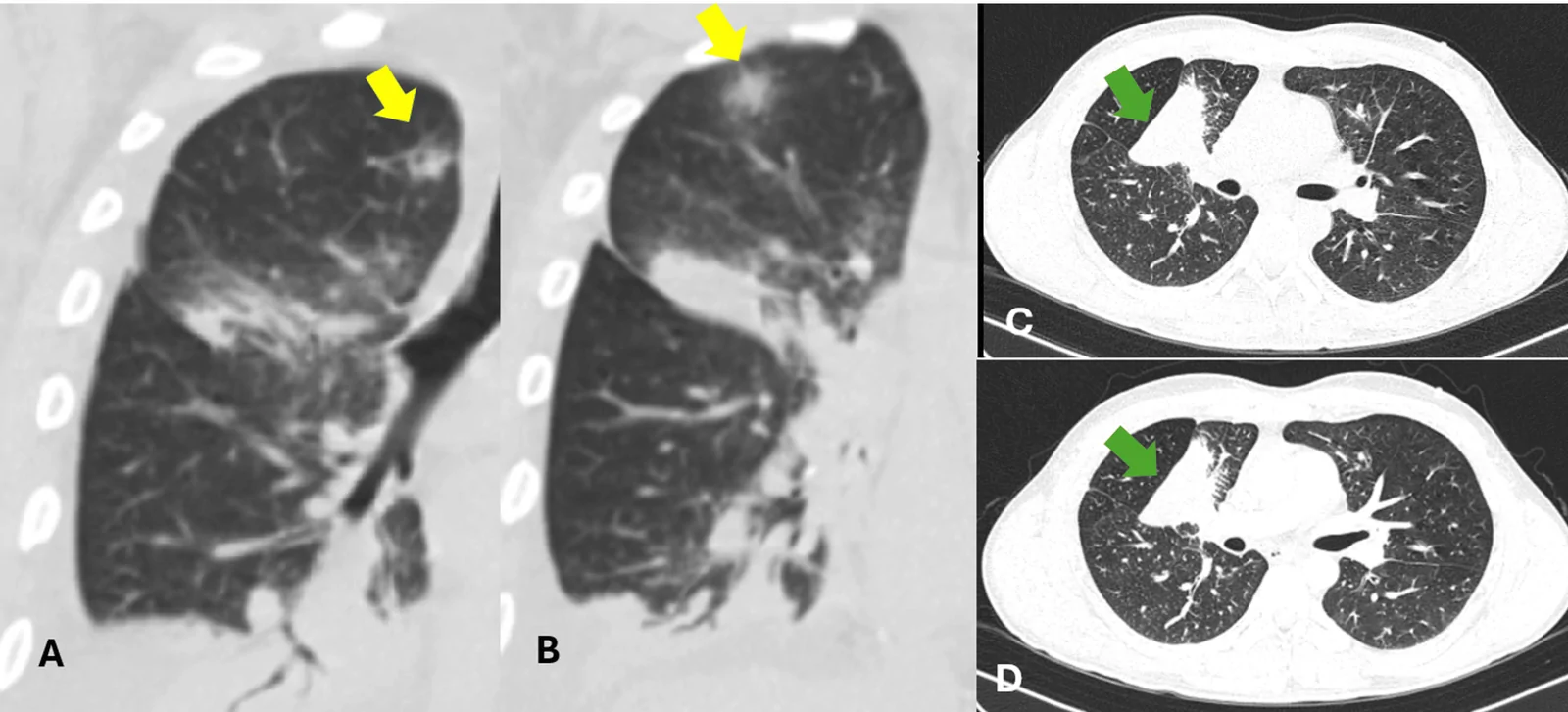
Chest computed tomography (CT) A–B (coronal reconstructions): Peripheral and subpleural ground-glass opacities are identified in the right upper lobe, with areas of mild confluence (yellow arrows). C (axial image at admission): Alveolar consolidation in the anterior segment of the right upper lobe, with ill-defined margins (green arrow). D (axial image after 14 days of treatment): Persistent alveolar opacity in the same region, with minimal change in extent and density, indicating limited radiological response to therapy (green arrow).

Chest radiography demonstrated a relatively dense opacity with a right parahilar atelectatic band and bilateral basal opacities, predominantly on the left. Chest computed tomography (CT) revealed bilateral pleural effusions, right apical consolidation, and ground-glass nodules (see Figure 1A-1C). Point-of-care ultrasound (POCUS) showed a hyperdynamic heart and a large pericardial effusion with right chamber collapse, consistent with cardiac tamponade physiology. Urgent pericardiocentesis drained 100 cc of hemorrhagic fluid with a lymphocytic exudative pattern (lymphocytes 69%) and markedly reduced glucose (16.3 mg/dL) - a finding associated with inflammatory or neoplastic pericarditis. Infectious workup was negative (Gram stain, bacterial and mycobacterial cultures); cytological examination for malignant cells was not performed. Left-sided thoracentesis was also performed, yielding results consistent with a lymphocytic exudate (pleural/serum protein ratio 0.59).

During hospitalization, pulmonology evaluation included bronchoscopy with bronchoalveolar lavage (BAL) to rule out infectious causes. Findings included acute bronchitis, markedly elevated galactomannan (7.99), negative molecular testing and culture for Mycobacterium tuberculosis, and cytology negative for malignancy. Based on these findings, infectious disease specialists considered pulmonary aspergillosis and initiated voriconazole therapy, with follow-up imaging planned in two weeks to assess treatment response.

Regarding acute kidney injury, nephrotoxic agents were discontinued, and renal ultrasound showed no evidence of obstruction or chronic structural abnormalities. Notably, renal function improved following pericardiocentesis.

During hospitalization, the patient developed marked asialia without spontaneous complaints of xerostomia or ocular symptoms. Schirmer's test showed severely reduced lacrimal secretion (3 mm right eye, 4 mm left eye). Minor salivary gland biopsy demonstrated preserved architecture without lymphocytic infiltration. Nonetheless, primary SS was initially considered because of positive antibodies and abnormal Schirmer's test results, despite a negative minor salivary gland biopsy, and immunosuppressive therapy was initiated with prednisone (40 mg/day), methotrexate (15 mg/week), colchicine (0.5 mg every 12 hours), and folic acid (1 mg/day).

**Figure 2:**
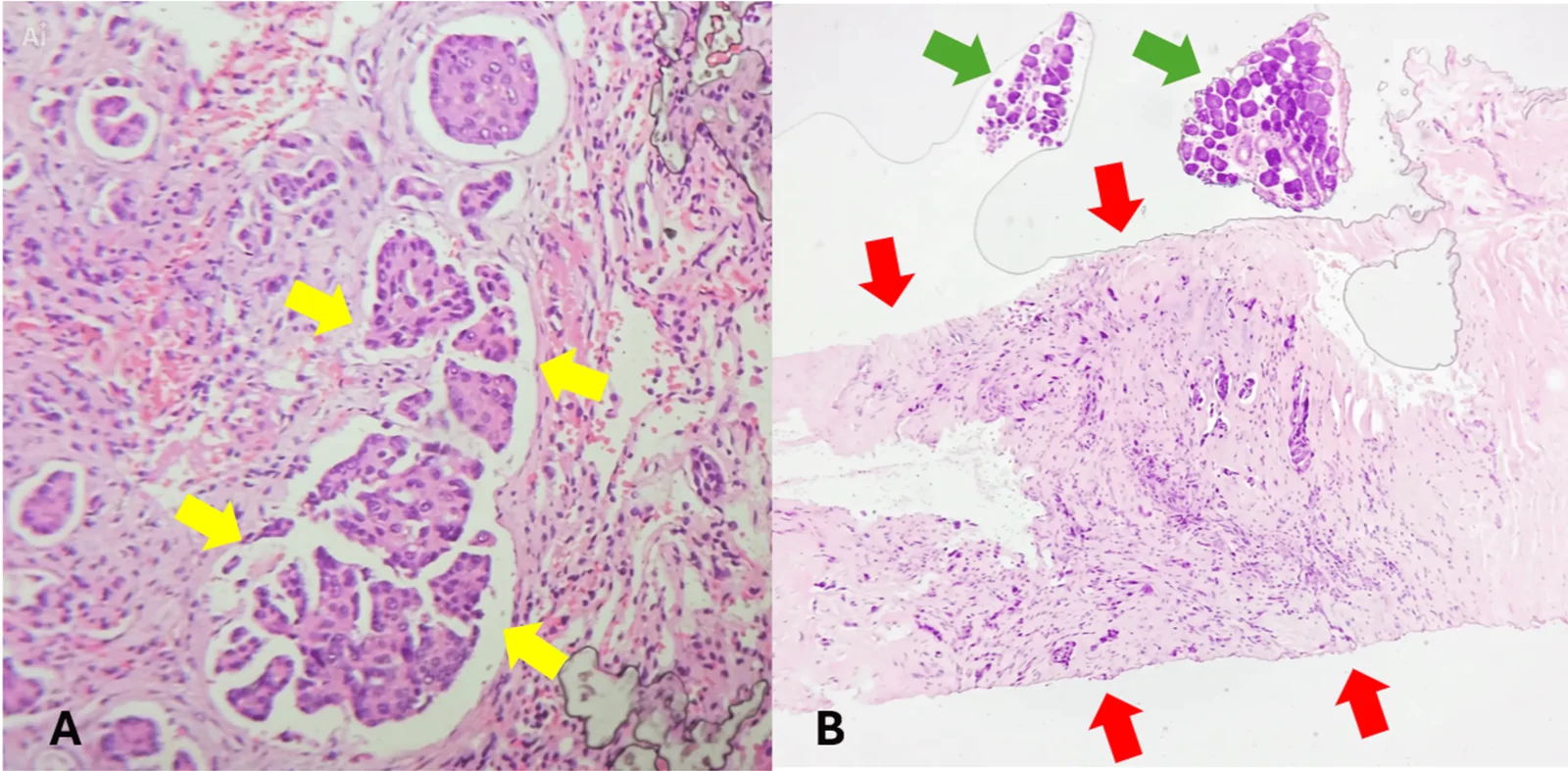
Photomicrographs of lung parenchyma and parotid gland (A) Right upper lobe lung parenchyma. Hematoxylin and eosin (H&E) stain, 10× magnification. Infiltration by a malignant neoplastic lesion with a micropapillary configuration is observed, composed of cells with abundant eosinophilic cytoplasm and hyperchromatic nuclei, some pleomorphic, elongated, and with pseudoinclusions. The cells are arranged in a diffuse nested pattern, associated with desmoplastic stroma and areas of necrosis (yellow arrows). (B) Left parotid gland. Hematoxylin and eosin (H&E) stain, 10× magnification. Histological sections show salivary gland fragments with preserved seromucous acinar architecture (green arrows), with adjacent connective tissue infiltrated by atypical tumor cells displaying a micropapillary configuration. These cells exhibit abundant eosinophilic cytoplasm and hyperchromatic nuclei, some pleomorphic, elongated, and with pseudoinclusions, arranged in a diffuse nested pattern and inducing a desmoplastic stromal reaction (red arrows).

Two weeks later, the patient was readmitted for follow-up contrast-enhanced chest CT, which showed persistent alveolar opacity in the right upper lobe (see Figure 1D). Given the persistent alveolar opacity and clinical suspicion of pulmonary aspergillosis, a joint decision by infectious disease and vascular surgery teams led to right upper lobectomy, which unexpectedly revealed invasive adenocarcinoma with micropapillary and solid patterns (see Figure 2A). Immunohistochemistry demonstrated a profile negative for primary lung origin, with positive staining for GATA-3, CK7, CKAE1/E3, and CK19, suggesting a salivary gland origin. A soft tissue ultrasound of the left parotid region identified a heterogeneous nodular lesion with internal calcifications (24 × 18 mm) and regional laterocervical lymphadenopathy. Biopsy of the lesion confirmed metastatic adenocarcinoma (see Figure 2B).

**Figure 3:**
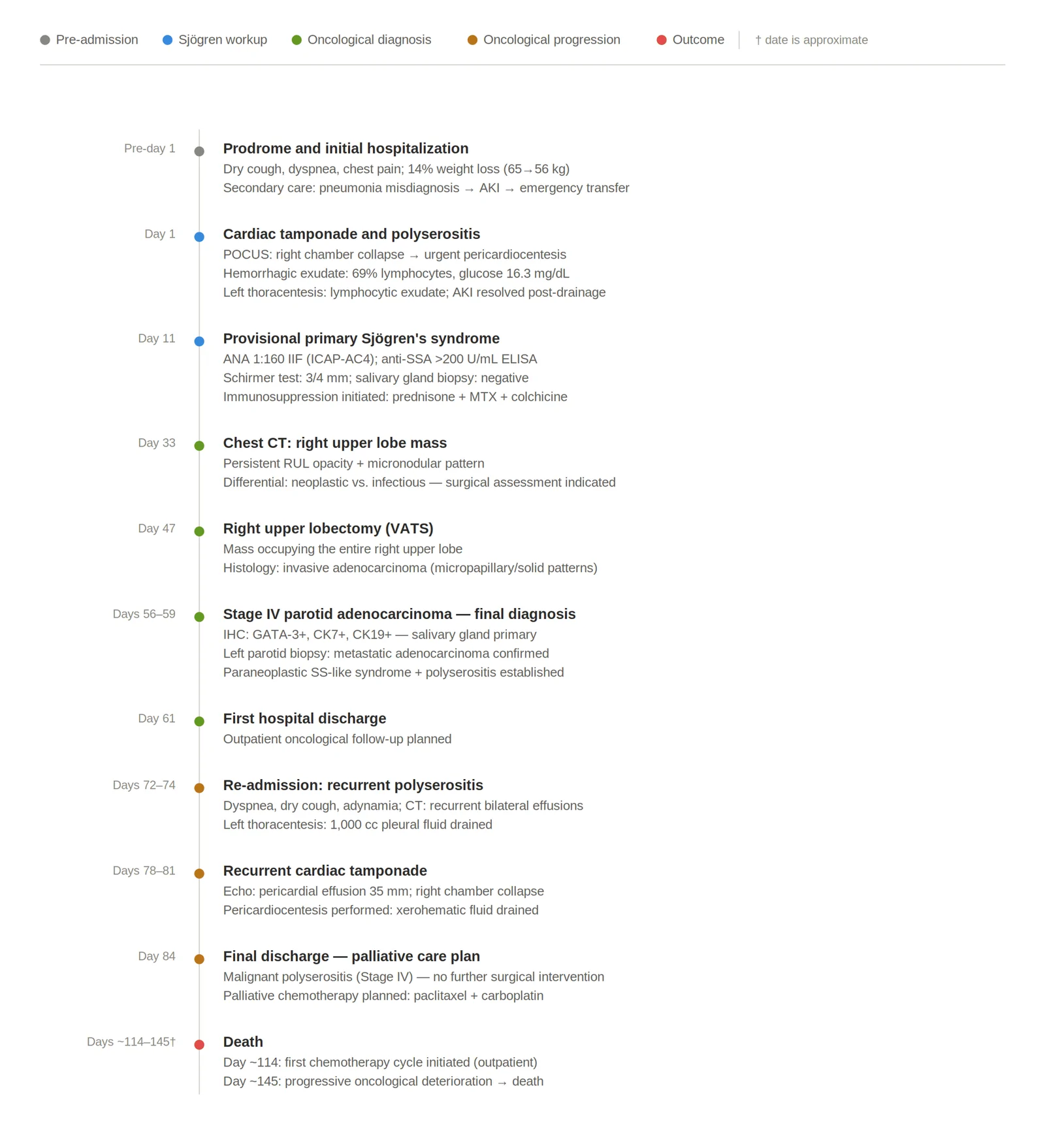
Clinical timeline of a 43-year-old male with stage IV left parotid adenocarcinoma and paraneoplastic Sjögren’s-like syndrome. The timeline depicts the 145-day clinical course across five phases. Gray: pre-admission prodrome and initial misdiagnosis as community-acquired pneumonia, complicated by AKI and emergency transfer. Blue: Sjögren’s syndrome workup and provisional diagnosis (days 1–11). Teal: oncological diagnosis phase (days 33–61), including right upper lobectomy, confirmatory immunohistochemistry, and parotid biopsy establishing stage IV left parotid adenocarcinoma with paraneoplastic Sjögren’s-like syndrome and polyserositis. Amber: second hospitalization for oncological progression with recurrent malignant polyserositis requiring drainage procedures (days 72–84). Coral: palliative chemotherapy and death (days ~114–145†). †Approximate, based on external information. AKI, acute kidney injury

Based on the clinical course and findings, a diagnosis of stage IV left parotid adenocarcinoma with pulmonary metastases, polyserositis (pericardial and pleural effusions), accompanied by a probable paraneoplastic syndrome with a Sjögren-like phenotype (Figure 3).

Shortly after the initial discharge, the patient was readmitted with recurrent dyspnea and dry cough, along with recurrent bilateral pleural effusions requiring thoracentesis. He subsequently developed recurrent cardiac tamponade, confirmed by echocardiography, which required a further pericardiocentesis. Given the recurrent, refractory nature of the malignant polyserositis, the patient was discharged with a palliative care plan.

The oncology team initiated urgent systemic chemotherapy with paclitaxel plus carboplatin, with palliative intent given the advanced stage and high tumor burden. The patient received only one cycle of chemotherapy; however, he subsequently experienced progressive clinical deterioration and died.

## Discussion

SS is a systemic autoimmune disease characterized by dysfunction of exocrine glands, the presence of autoantibodies-particularly anti-Ro/SSA-and, in some cases, extraglandular involvement with an increased risk of lymphoma [1,2]. Both classical and contemporary literature recognize that primary SS represents a diagnostic challenge due to its well-established association with lymphoproliferative complications, with lymphoma developing in approximately 2-5% of patients, which justifies a low threshold for malignancy screening [3]. By contrast, Sjögren-like paraneoplastic syndromes associated with solid tumors are uncommon and are mainly described in isolated reports, with marked clinical heterogeneity, variable temporal relationships, and the absence of a consistent serological pattern [4].

Our case illustrates an initial presentation of severe polyserositis, persistent pulmonary involvement-including lesions unresponsive to antimicrobial therapy-and constitutional symptoms, such as weight loss, initially interpreted as SS, which ultimately corresponded to stage IV parotid adenocarcinoma with pulmonary metastases, consistent with a paraneoplastic Sjögren-like phenotype. In this context, the discordance between marked serological positivity (anti-SSA/Ro, abnormal Schirmer test) and the absence of focal sialadenitis is particularly relevant, highlighting the need to broaden the differential diagnosis in the presence of atypical systemic manifestations, rapid progression, and lack of response to conventional therapy.

SS as a paraneoplastic phenomenon is an exceptionally rare entity described in oncologic patients. In large series of malignancies associated with paraneoplastic syndromes, SS is uncommon, with reported frequencies below 1% (≈0.19% in lung cancer and 0.9% in myelodysplastic syndromes) [5]. The first reported case of paraneoplastic Sjögren-like syndrome identified in the literature was described by Janssen et al. in a patient whose SS preceded small cell lung carcinoma (“oat cell carcinoma”) [6]. In our review, 14 additional cases were identified, summarized in 11 reports (Table 2), one of which corresponds to a case series of five patients.

**Table 2:** Comparative summary of reported cases of paraneoplastic Sjögren's syndrome (11 reports comprising 14 individual cases, plus the present case) ANA: antinuclear antibodies, ENA: extractable nuclear antigens, anti-SSA (Ro): Sjögren's syndrome-related antigen A, anti-SSB (La): Sjögren's syndrome-related antigen B, C3/C4: complement components, SCLC: small cell lung cancer, LEMS: Lambert–Eaton myasthenic syndrome, FS: focus score, MTX: methotrexate, HCQ: hydroxychloroquine, EOX: epirubicin, oxaliplatin, capecitabine Note: Entry 7 corresponds to a case series of five patients (Dakar, Senegal) reported as a single entry; thus, the 11 entries in this table collectively represent 14 previously reported cases plus the present case (entry 11).

Case	Author / year / country	Sicca manifestations	Systemic manifestations	Schirmer test	Glandular biopsy	Neoplasm / stage (metastasis)	Clinical course and treatment
1	Nishimura / 2006 / Japan [7]	Xerostomia, xerophthalmia, markedly reduced tear production	LEMS: severe lower limb weakness, difficulty walking	Positive (reduced tear secretion)	Positive: periductal inflammatory cell aggregates (labial gland)	Lung cancer (SCLC) / IIIA (hilar and mediastinal nodes)	Chemoradiotherapy reduced tumor; LEMS improved; xerostomia only mildly improved
2	Khamitov / 2018 / Russia [8]	Xerostomia, facial edema due to parotid enlargement	Polyarthritis, fever (37.5°C), weight loss	Not specified	Not performed	Gastric cancer / T1bN1M0 (2/13 regional nodes)	Gastrectomy + chemotherapy (EOX). Complete resolution of sicca, arthritis, and fever
3	Ali & Mahto / 2020 / UK [9]	Oral sicca syndrome (dry oropharyngeal mucosa)	Trigeminal neuropathy: facial numbness, fatigue, facial allodynia	Negative	Not performed	Ovarian cancer / metastatic (mediastinal nodes, peritoneal deposits)	Referred for palliative chemotherapy
4	Bouomrani / 2025 / Tunisia [5]	Xerostomia and xerophthalmia	Seronegative polyarthritis, left parotid swelling	Positive (dry eye confirmed)	Positive: chronic lymphocytic sialadenitis (Focus score = 1)	Parotid carcinoma (undifferentiated) / advanced (regional lymph nodes)	Radical parotidectomy + radiotherapy. Complete resolution; antibodies negative at 3 months
5	Smith (C1) / 2023 / Australia [10]	Severe xerophthalmia	Polyserositis: acute pericarditis, pleural effusion, colonic serositis	Severe: 1 mm/5 min	Not performed	Thymic carcinoma / metastatic (pleura, bone)	Declined chemotherapy; managed conservatively with pericardial window
6	Smith (C2) / 2023 / Australia [10]	Xerophthalmia, occasional oral ulcers	Fatigue, recurrent sinopulmonary infections	Not performed (clinically confirmed)	Not performed	Thymic carcinoma / metastatic (liver, lungs, bone, nodes)	Chemoimmunotherapy (carboplatin/paclitaxel/atezolizumab); good tumor response; developed lupus flare
7	Domingues / 2012 / Portugal [11]	Chronic xerostomia and xerophthalmia	Sensory neuronopathy: severe ataxia, paresthesia	Positive (reduced tears)	Positive: focal mononuclear infiltrate (>50 cells/4 mm³)	Breast cancer / metastatic (axillary and supraclavicular nodes)	Mastectomy + chemotherapy; no neurologic improvement
8	Oliveira / 2021 / Portugal [12]	Severe xerostomia and xerophthalmia	Symmetric polyarthritis, 15 kg weight loss, stroke	Positive (severe dry eye)	Positive: focal sialadenitis (1 focus/4 mm²)	Pancreatic cancer / metastatic (liver, hilar nodes, lung)	Carboplatin + paclitaxel; partial improvement; death due to progression
9	Dakar series / 2023 / Senegal [13]	Universal (100%): lacrimal, salivary, vaginal involvement	Polyarthritis (80%), portal hypertension (20%), chronic diarrhea (20%)	Not specified (clinically confirmed)	Positive: ≥Grade 3 sialadenitis (100%)	Prostate (3), ovary (1), colon (1) / (liver, peritoneum, lung)	MTX, HCQ + chemotherapy; poor prognosis; 40% mortality due to metastasis
10	Bartoloni / 2012 / Italy [14]	Oral and ocular sicca syndrome	Symmetric arthritis (wrists, small joints)	Positive	Non-specific: mild mononuclear infiltrate	Renal cancer / Grade II (non-capsular)	Nephrectomy; complete resolution; antibodies negative
11	Berrocal et al. / 2026 / Colombia	Xerostomia, foreign body ocular sensation	Polyserositis: cardiac tamponade, pleural effusion; 14% weight loss	Positive: 3 mm (right), 4 mm (left)	Negative: preserved architecture, no lymphocytic infiltration	Parotid adenocarcinoma / Stage IV (micropapillary lung metastasis)	Paclitaxel + carboplatin; poor outcome with subsequent death

Current reports demonstrate notable heterogeneity, including carcinomas of the lung [7], stomach [8], ovary [9], thymus [10], breast [11], pancreas [12], colon [13], prostate [13], and kidney [14]. This entity typically occurs in older patients (mean age 73.4 years) and may precede tumor diagnosis in approximately two-thirds of cases, although synchronous presentation is a hallmark of its paraneoplastic nature. Clinically, it may present in a complete or incomplete form, or even as isolated autoimmune xerosis characterized by xerostomia and/or xerophthalmia without evident systemic involvement [7]. Furthermore, paraneoplastic SS may coexist with other paraneoplastic manifestations, including subacute sensory neuropathy or Lambert-Eaton myasthenic syndrome, underscoring the complexity of its clinical recognition [7]. Reports by Bouomrani et al. [5] and Smith et al. [10] consistently indicate that recent onset, rapid progression of xerosis, significant weight loss, and particularly poor response to standard immunosuppressive therapy are suggestive of an underlying occult malignancy.

A defining criterion of paraneoplastic origin is the complete resolution of clinical and immunological features following radical treatment of the tumor, as documented in cases reported by Khamitov (gastric) [8], Bouomrani (parotid) [5], and Bartoloni (renal) [14].

Our case differs from previously reported cases in that, while most series describe pathological salivary gland biopsies, our patient's biopsy was negative. This absence of lymphocytic infiltration suggests that the paraneoplastic syndrome may manifest as predominantly functional glandular dysfunction mediated by autoantibodies, without the chronic tissue destruction typical of primary SS. Alternatively, this presentation may represent an immunological epiphenomenon secondary to aberrant tumor antigen presentation, whereby ectopically expressed tumor antigens trigger immune cross-reactivity with salivary gland self-antigens - a mechanism that has been proposed as a unifying pathophysiological explanation for autoimmune-mediated paraneoplastic syndromes [15]. Both hypotheses are not mutually exclusive and may act in concert, underscoring the complexity of immune dysregulation in the setting of advanced malignancy.

Unlike most reports of paraneoplastic Sjögren-like syndrome describing arthritis or fatigue, this case is characterized by severe polyserositis with pericardial effusion and cardiac tamponade physiology. Although pleural and pericardial effusions have been exceptionally reported in primary SS [16,17,18], they remain rare extraglandular manifestations; in our patient, the severity of serositis, rapid progression, and lack of response to immunosuppressive therapy were more consistent with malignancy-associated serositis than with primary SS. Pericardial fluid analysis revealed a hemorrhagic lymphocytic exudate with markedly reduced glucose (16.3 mg/dL). Although low pericardial glucose has classically been associated with infectious etiologies, both excluded by negative cultures and smear, studies have shown that pericardial glucose ≤70 mg/dL is significantly associated with malignancy-related effusion, with a specificity of 89.6% and a positive predictive value of 85.7% [19]. In addition, cardiac tamponade physiology, large effusion volume, and hemorrhagic appearance are recognized high-risk features more consistent with neoplastic than autoimmune pericardial disease [20]. Cytological examination was not performed, which represents a recognized limitation of this case.

The history of smoking in this patient represents an established risk factor for parotid gland tumors. Epidemiological evidence demonstrates a statistically significant association between cigarette smoking and parotid gland neoplasms, with an odds ratio of 1.66 (95% CI, 1.31-2.11) reported in a nationwide case-control study [21,22]. Although this association is of lesser magnitude than that observed for lung cancer, a lower odds ratio does not negate the existence of a real and demonstrated risk.

The markedly elevated galactomannan in bronchoalveolar lavage (7.99) supported a probable coexisting pulmonary aspergillosis in areas of structural lung involvement [23,24]. Although histopathological confirmation was lacking, this does not exclude the diagnosis, as negative tissue findings can result from focal hyphal distribution, sampling error, or prior antifungal therapy, which reduces fungal burden and histological sensitivity [25,26].

This report has several limitations. First, a definitive causal relationship between the paraneoplastic Sjögren-like syndrome and the underlying malignancy cannot be established, and the association remains inferential. Second, the absence of histopathological confirmation of pulmonary aspergillosis limits diagnostic certainty despite strong microbiological evidence; additionally, prior antifungal therapy may have reduced the sensitivity of tissue-based diagnostic methods. Third, cytological examination of the pericardial fluid was not performed, precluding definitive exclusion of direct neoplastic pericardial involvement. Finally, the patient's early death precluded evaluation of clinical evolution following oncologic treatment, limiting confirmation of the paraneoplastic nature through symptom resolution. Notably, the patient fulfilled the 2016 ACR/EULAR classification criteria for primary SS; however, these criteria were developed for disease classification rather than for establishing causality or distinguishing paraneoplastic mimics.

Regarding strengths, this case describes a rare paraneoplastic Sjögren-like presentation associated with a salivary gland malignancy, notable for severe polyserositis and strong serological positivity in the absence of lymphocytic infiltration on salivary gland biopsy. Although the patient fulfilled the 2016 ACR/EULAR classification criteria for primary SS, the rapid clinical course, severe constitutional symptoms, negative salivary gland biopsy, poor response to immunosuppression, and concurrent metastatic malignancy favored a probable paraneoplastic Sjögren-like syndrome rather than primary disease. It should be acknowledged, however, that smoking per se can induce salivary gland dysfunction and reduce glandular cellularity, potentially contributing to a false-negative biopsy result independently of the autoimmune or paraneoplastic process; this represents an additional confounding factor in the histological interpretation. The case further integrates clinical, serological, and oncological findings in a complex diagnostic context and provides a structured synthesis of previously reported cases.

## Conclusions

Paraneoplastic Sjögren-like syndrome is a rare but clinically relevant entity that may present without classic histopathological features, including lymphocytic infiltration on salivary gland biopsy. This case supports the notion that paraneoplastic glandular dysfunction may be predominantly antibody-mediated rather than driven by direct tissue destruction, and that a negative biopsy alone should not exclude a probable paraneoplastic etiology. As the patient's early death precluded assessment of clinical response to oncologic treatment, the paraneoplastic nature remains probable rather than confirmed. A high index of suspicion for underlying malignancy is warranted in atypical autoimmune presentations with rapid progression or poor response to immunosuppression, as early recognition may improve diagnostic timeliness and prognosis.
